# Treating maternal depression: understanding barriers and facilitators to repetitive transcranial magnetic stimulation treatment in Canada-a protocol

**DOI:** 10.3389/fpsyt.2023.1143403

**Published:** 2023-07-27

**Authors:** Huda F. Al-Shamali, Margot Jackson, Nataliia Zinchuk, Setayesh Modanloo, Gina Wong, Bo Cao, Lisa Burback, Xin-Min Li, Andrew Greenshaw, Yanbo Zhang

**Affiliations:** ^1^Department of Psychiatry, University of Alberta, Edmonton, AB, Canada; ^2^Department of Nursing, University of Alberta, Edmonton, AB, Canada; ^3^Faculty of Health Disciplines, Athabasca University, Athabasca, AB, Canada

**Keywords:** peripartum depression, repetitive transcranial magnetic stimulation, treatment, experiences, barriers, facilitators

## Abstract

**Background:**

Peripartum depression (PPD) is a serious public health issue associated with severe and potentially long-term adverse maternal and child developmental outcomes. Suicide and overdose, for example, accounts for up to a third of maternal deaths. A current depression diagnosis with no active treatment is a common risk factor for maternal suicide. Repetitive transcranial magnetic stimulation (rTMS) is a non-pharmacological treatment that has recently shown some promise as an effective treatment with limited side effects for PPD, but more research is required. This study aims to identify current barriers and potential facilitators for women with PPD accessing treatment in general, and rTMS specifically.

**Methods:**

This study will consist of two anonymous, self-administered surveys, focus groups, and interviews. A descriptive interpretative approach will be employed, and thematic analysis will be completed for the focus groups and interviews. Participants who are currently, or have previously experienced depressive symptoms, as well as health providers will be recruited. Our study will follow an equity, diversity, and inclusion (EDI) perspective on sex, gender, and ethnicity and the gender-based analysis plus (GBA+) analytic tool will be used. Both a qualitative and quantitative analysis of the data will be conducted.

**Discussion:**

We expect to find education and accessibility to be primary treatment barriers for persons with PPD. Identifying and addressing barriers is a critical first step towards the devolvement of initiatives that can work towards improving mental health in this population.

## Introduction

Peripartum depression (PPD) is a serious and often persistent disorder that negatively impacts the mother and child’s short-term and long-term health outcomes and presents a heavy societal burden (1). PPD refers to a major depressive episode with onset during pregnancy or within the first 4 weeks after delivery (2). Patients often experience persistent sadness and lack of interest, sleep and appetite disturbances, suicidal ideations and attempts, and difficulties in thinking and concentration (2).

Women suffering from PPD may experience worsening physical health (i.e., worse aerobic capacity) (1), increased healthcare utilization (1), worsening psychological health (i.e., increased anxiety, long-term depression, lower self-esteem, stress) (1, 3), lower quality of life (1, 4), impaired relationships (i.e., lower social functioning and support, distant and difficult partner relationships) (1), increased risk-taking behavior (i.e., smoking) (1, 5), and increased suicidal ideation with suicide being responsible for 8%–30% of maternal death (1, 6, 7).

Mother-infant bonding is also negatively impacted by PPD. A 2019 systematic review found that mothers with PPD were less emotionally available, warm, and sensitive toward their children (1). This withdrawn behavior and limited attachment can translate to poor child physical health (i.e., prolonged illness with diarrhea, increased pain response, more instances of night-time awakening) and impaired cognitive, social, language, emotional, and behavioral development (1). Another point of concern is the increased rate of mortality in this population of children. It’s been observed that probable postpartum depression was linked to a three-fold increase in the risk of infant death during the first 6 months after delivery and a two-fold increase during the first 12 months (8).

PPD is often treated through psychotherapy, pharmacotherapy, and electroconvulsive therapy (ECT) (9). Psychotherapy is often used to treat mild to moderate depression but demonstrates limited effectiveness when treating more severe and treatment-resistant forms of depression (10, 11). Pharmacotherapy is typically added to psychotherapy to increase treatment effectiveness (12). However, taking medication while pregnant or breastfeeding is considered unacceptable by many patients due to the potential effects it may have on their child (i.e., neonatal distress, preterm birth) (13, 14).

ECT is effective in treating resistant and life-threatening depression and is considered safe for use during pregnancy by the American Psychiatric Association and the American College of Obstetricians and Gynecologists (15). Yet, the surrounding stigma and its side effects (i.e., nausea, memory problems, muscle pain) limit the desirability and perceived acceptability of ECT treatment (16, 17). Additionally, receiving ECT while pregnant is associated with an increased risk for aortal compression and aspiration in the mother, and possible fetal heart rate changes (16).

There is a growing need for safe and effective treatments for persons suffering from PPD as evinced by the rising suicide rate, the constant rate of depression, the increasing disability associated with depression, and the limited acceptable treatment options available for this population (2, 3, 10–18).

Over the last two decades, repetitive transcranial magnetic stimulation (rTMS) has emerged as a promising effective and safe treatment option for treatment-resistant depression (TRD) (19, 20). rTMS is a non-pharmacological treatment that uses magnetic field pulses to elicit electrical current changes in the dorsolateral prefrontal cortex (DLPFC) (21). There is evidence that rTMS exerts antidepressant effects by causing recurrent and consistent firing of coactive cortical neurons, thereby potentiating plasticity in the cortex (22). Additionally, meta-plasticity, or the plasticity of synaptic plasticity, is thought to be modulated by non-invasive brain stimulation techniques such as rTMS and may also play a role in its therapeutic effects in depression (23). In 2002 and 2008, rTMS received approval from Health Canada and the US Food and Drug-Administration (FDA), respectively, as a treatment for TRD.

More recently, rTMS has been investigated as a treatment for PPD with onset during pregnancy (24–26) and during the postpartum period (27–30). Although limited, the current literature suggests that rTMS is an effective and safe treatment with minimal side effects. However, due to the small sample sizes and the paucity of randomized controlled trial data, more research is needed to further delineate the effectiveness of rTMS on patients with PPD.

Despite the availability and safety of rTMS as a treatment for PPD, there is a limited uptake in this population which begs the question “why aren’t more women with PPD receiving rTMS?” Understanding facilitators to treatment and barriers preventing women with PPD from accessing rTMS treatment is the first step toward ensuring that everyone can access safe and effective treatments. The limited literature assessing the knowledge, experiences, and attitudes of psychiatrists (31, 32), patients with depression (33), and pregnant women (34) towards rTMS treatment found insufficient knowledge and negative attitudes. More research is needed to identify current barriers and potential facilitators for women with PPD accessing treatment in general, and rTMS specifically. We expect to find insufficient knowledge on how to access mental health treatment during the peripartum period, limited awareness of the existence of rTMS treatment, and accessibility related concerns.

## Methods

### Objectives and purpose

Our research aims to identify barriers preventing women with PPD from accessing treatment generally, and rTMS treatment specifically. This will be accomplished by engaging with mental health professionals, adults who experienced depressive symptoms during the peripartum period, and adult members of their families. By connecting with these communities, we strive to better understand the concerns of participants and ascertain potential avenues for improving mental health service access and delivery. Our study will follow an equity, diversity, and inclusion (EDI) perspective on sex, gender, and ethnicity and the gender-based analysis plus (GBA+) analytic tool will be used.

### Ethics

This study received ethical approval from the University of Alberta Research Ethics Office (Pro00114151). Informed consent will be received from all participants. All participants will read through a consent form prior to the start of the study. Participants completing the survey will then select yes/no to four questions confirming they are 18 years or older, read and understood the information form, and consent to participating in the study. Prior to the start of the focus group, members of the research team will ensure everyone has read and understood the consent form and be reminded that their participation implies their consent. Participants in the interviews will give verbal informed consent during the recorded and transcribed interview. This consent procedure has been reviewed and approved under Pro00114151.

### Trial design

#### Participant recruitment

Participants will be recruited from multiple sources on both a local and national scale. Locally, recruitment posters will be hung at women’s health clinics, family doctor offices, pediatrician offices, psychologist or therapist offices, rTMS clinics, and universities. We will also reach out to local organizations to establish a partnership for recruitment (i.e., Canadian Mental Health Association, Alberta Council of Women’s Shelters, Terra Centre).

For national recruitment, we are partnered with the Mood Disorders Society of Canada who will share our surveys with their network. Additionally, we will recruit participants nationally through advertised social media posts (i.e., Instagram).

#### Equity, diversity, and inclusion

All participants will be asked to provide the following demographic information: gender, age, ethnicity, and the first three values of their postal code. This information will allow us to perform EDI based analyses assessing the impact these identity factors have on a person’s access to depression treatment during the peripartum period.

Our survey is translated by RCIC Certified Document Translation and Translators into several languages (i.e., French, Arabic, Mandarin, Punjabi) to increase its accessibility and allow us to explore culture-linked barriers/facilitators to treatment from an EDI perspective. To ensure the meaning of the survey questions is preserved through the translation process, the translated surveys were reviewed for accuracy and consistency by native language speakers. Survey translation allows for the capture of multiple viewpoints and aids in the inclusion of ethnically diverse populations.

#### Gender-based analysis plus

The GBA+ tool was created by the Canadian Government to aid in the establishment of initiatives that are equitable, diverse, and inclusive of the cultural mosaics that shape our communities. The GBA+ analytic process can be divided into five major steps: (1) identify the issue, (2) challenge assumptions (unconscious bias), (3) gather facts, research, and consult, (4) develop options and make recommendations, and (5) monitor and evaluate. The GBA+ analytic tool informed our study design and will be used as we assess our findings and create recommendations for initiatives to improve patients with PPD’s access to treatment.

#### Survey

We will conduct two national anonymous, self-administered, five-minute online surveys shared through REDCap (35). REDCap is an electronic data capture tool hosted and supported by the Women and Children’s Health Research Institute at the University of Alberta. The literature was reviewed by research team members for pertinent survey questions assessing participants’ experiences, attitudes, and recommendations for rTMS. Then two surveys were created, through group discussion and consensus, that are an amalgamation of the search results and the research team’s insight. The survey assesses participants’ experiences with, and barriers to, rTMS treatment through a mix of multiple-choice questions and rating scales. One of the surveys will be completed by health professionals (i.e., primary care physicians, therapists, psychiatrists, psychologists, residents, and nurses), and the other will be completed by adults who experienced, or are experiencing, depressive symptoms during the peripartum period and their families (see Supplementary materials for survey questions. The survey can be shared and completed through this link: https://redcap.link/a8kchdcn).

Since there are no similar previously validated surveys, we underwent a one-month “trial period” where we began recruitment for the English version of the survey to assess its performance and make any necessary modifications prior to the release of the multi-language survey on a larger scale.

We completed an *a priori* power analysis through the G*Power 3.1 software and used an effect size of 0.15, an alpha error probability of 0.05, and a power of 0.95 (36). Our first survey directed to health professionals contains 10 predictors/variables and would require a minimum sample size of 172 participants. Our second survey directed to persons who experienced depressive symptoms during the peripartum period, and their families contains 15 predictors/variables and would require a minimum sample size of 199 participants. The survey will collect demographic and clinical data. Categorical data will be statistically analyzed using the chi-square test, numerical data will be analyzed using the multivariate analysis of variance (MANOVA), and continuous variables will be analyzed using independent and paired *t*-tests. All data analysis will be conducted using the Statistical Package for the Social Sciences (SPSS^®^; IBM^®^ SPSS^®^ Statistics Premium for Macintosh, Version 28.0, IBM Corp., Armonk, NY, United States).

#### Focus groups

Three focus groups will be conducted over ZOOM to discuss barriers and facilitators to rTMS treatment and recommendations for improving accessibility. One focus group will be with psychiatrists who conduct rTMS treatment, the second with PPD patients, and the third with general practitioners. Each focus group will enroll 8–10 participants. The 60 min focus groups will be automatically transcribed by ZOOM, then reviewed for errors and anonymized by a researcher. A descriptive interpretative approach will be employed, and thematic analysis will be completed using the NVivo software.

#### Interviews

Twenty participants will undergo a 30 to 60 min one-on-one key informant semi-structured interview. The interviews will allow us to more deeply understand the facilitators of treatment and the barriers preventing patients with PPD from receiving treatment generally and rTMS specifically. In addition, it will allow for a discussion on improvements that can be made to improve the mental treatment access process (see Supplementary materials for Interview Guiding Questions). An interpretative descriptive approach will be employed. Interviews will be conducted through ZOOM with audio and video recordings. An automatic transcription of the interview will be produced by ZOOM and reviewed for accuracy by a researcher. Participants can opt for an in-person interview if they prefer. In-person interviews will be audio-recorded and manually transcribed by a member of the research team. Transcripts will be anonymized prior to the completion of the analysis. A thematic analysis will be completed using the NVivo software. The interview will consist of inductive open-ended questions. Interviews will be conducted until we reach a point of data saturation as detailed in Braun and Clarke (37).

## Discussion

Receiving timely and effective mental health treatment is imperative for the health of expectant and new mothers and their children (38). Yet, many barriers exist preventing this population from accessing the care they need. rTMS presents a possible treatment avenue, but more research is needed to understand what barriers and facilitators are impacting women with PPD’s access to mental health treatment. We expect to find that awareness of what treatment options are available and an individual’s ability to access them will be major barriers, especially for rTMS treatment (31–34). These barriers were observed in a 2011 study by Kim and colleagues that asked 500 pregnant women to select from a list of treatments which they considered acceptable to receive. Only 0.2% selected magnetic therapy (rTMS). This number went up to 15.7% in participants who watched an information video on rTMS first. This study also identified the most common treatment barriers to be transportation, work schedule, and a belief that symptoms will improve on their own (34). This study is a great start, but more research is needed to fully understand all the factors that impact women with PPD from accessing treatment at every stage of the treatment access process (i.e., seeking help, referral process, receiving treatment). In addition, there is a need for an EDI-based analysis that can begin to shed light on any differences across the different populations of responders. This study acts as the information gathering step that will help inform future initiatives that can begin to address the identified barriers and help realize a future where everyone can better access the mental health care they need.

### Limitations

The online nature of our study presents a selection bias as not everyone has equal access and competency with online platforms (i.e., REDCap, ZOOM). To mitigate this, members of the research team will visit several local organizations and provide the opportunity and the means for eligible participants to complete the survey and present the option to conduct an in-person interview. A second limitation is the potential for recall bias. Since the various components of the study asked participants about past experiences with PPD treatment, it is possible that a participant may not accurately recall details of the experience or may be inclined to exaggerate or understate their experience. Finally, it is important to note that the proposed study will be conducted exclusively in Canada. Since a potential barrier to accessing rTMS treatment may be related to systemic and reimbursement-related issues, the generalizability of the findings may be limited. The Canadian healthcare system is primarily publicly funded with much of its services concentrated in urban areas. As such, the findings of our research may not fully capture the barriers and facilitators that would exist in a country that is more densely populated and/or fully relies on a private healthcare system, such as the United States of America (USA). However, due to the multiple avenues available to receiving rTMS in a Canadian context, this limitation is minimized. Although Canadian healthcare primarily follows a single-payer system, rTMS can also be accessed through private clinics that require out-of-pocket payments or can be partially or fully covered by private insurance plans, mimicking the characteristics of a private healthcare system.

## Ethics statement

This study received ethical approval from the University of Alberta Research Ethics Office (Pro00114151). Informed consent will be received from all participants. Participants in the survey and focus group will give implied consent, while participants in the interviews will give verbal consent. This consent procedure has been reviewed and approved under Pro00114151.

## Author contributions

HA-S, YZ, MJ, NZ, SM, GW, and AG aided in the study design. HA-S wrote the protocol. HA-S, YZ, MJ, NZ, SM, GW, BC, LB, X-ML, and AG read and edited the manuscript. All authors contributed to the article and approved the submitted version.

## Funding

The article publishing charge was funded by a start-up fund provided by the Faculty of Medicine and Dentistry at the University of Alberta to YZ.

## Conflict of interest

The authors declare that the research was conducted in the absence of any commercial or financial relationships that could be construed as a potential conflict of interest.

## Publisher’s note

All claims expressed in this article are solely those of the authors and do not necessarily represent those of their affiliated organizations, or those of the publisher, the editors and the reviewers. Any product that may be evaluated in this article, or claim that may be made by its manufacturer, is not guaranteed or endorsed by the publisher.
