# Supplementary material for: Treating maternal depression: understanding barriers and facilitators to repetitive transcranial magnetic stimulation treatment in Canada-a protocol
Source: Front Psychiatry. 2023 Jul 27;14:1143403. doi: 10.3389/fpsyt.2023.1143403 (PMC10412871; doi:10.3389/fpsyt.2023.1143403)
Supplement: Supplementary file 1 [file Data_Sheet_1.docx]

**SURVEY**

1. Are you a healthcare provider (yes or no)
   1. *If no,*
      1. Which of the following applies to you (select all that apply):

1. I am currently diagnosed with peripartum depression

2. I was previously diagnosed with peripartum depression

3. I felt depressed while pregnant or shortly after giving birth

4. I am a family member of someone who is currently, or was previously, diagnosed with peripartum depression

5. None of the above

**Participants who answer “yes” to question 1 will be directed to Survey 1: Healthcare Providers. Participants who answer “no” to question 1 and select choices “1-4” under question i will be directed to Survey 2: Patients with peripartum depression or their family. The survey will automatically end for participants who choose option “5” under question i.**

________________________________________________________________________

**Survey 1: Healthcare providers**

*Demographic*

1. What gender do you currently identify as
   1. Woman
   2. Man
   3. Non-binary
   4. Transgender
   5. Prefer not to say
   6. Other (please specify)
2. What is your age (in years)?
3. Please specify your ethnicity. (Select all that apply)
   1. Asian/Pacific Islander
   2. Latino or Hispanic
   3. African/Caribbean/Black Canadian
   4. Caucasian
   5. First Nations/Metis/Inuit
   6. Middle Eastern
   7. Unknown
   8. Prefer not to say
   9. Other (please specify)
4. What are the first three digits of your workplace postal code?
5. What is your professional role?
   1. Primary care physician
   2. Therapist
   3. Psychiatrist
   4. Psychologist
   5. Resident
   6. Nurse practitioner
   7. Registered nurse
   8. Social worker
   9. Other (please specify)
6. What is your main place of work?
   1. General hospital
   2. Psychiatric hospital
   3. Private Clinic
   4. Other (please specify)

_ _ _ _ _ _ _ _ _ _ _ _ _ _ _ _ _ _ _ _ _ _ _ _ _ _ _ _ _ _ _ _ _ _ _ _ _ _ _ _ _ _ _ _ _ _ _ _ _ _

1. Have you ever provided professional services or treatments to people suffering from depression? *(answer options: Yes or No)*
   1. *If answer yes,*
      1. Which treatment(s) do you provide? (Select all that apply)
         1. Repetitive Transcranial Magnetic Stimulation (rTMS)
         2. Psychotherapy
         3. Pharmacotherapy
         4. Electroconvulsive Therapy (ECT)
         5. Other (please specify)
            1. *If select rTMS in question 1.i*

How accepting were your patients of the prospect of receiving rTMS treatment? *(Will answer on a scale of 1-10 with 1 being Extremely Accepting and 10 being Very Unaccepting)*

Would you treat someone with peripartum depression with rTMS?

*If no,*

What is preventing you from treating a person with peripartum depression using rTMS? (Select all that apply)

Its effectiveness

Safety concerns

Patient acceptance

Access to treatment

Time-consuming treatment

Cost

Other (please specify)

*If yes,*

What encourages you to treat a person with peripartum depression using rTMS? (Select all that apply)

Its effectiveness

Limited side effects

Patient acceptance

Easily accessible

Time efficient treatment

Cost

Other (please specify)

1. Have you ever referred patients to receive further treatment for their depression outside of your office? *(answer options: Yes or No)*
   1. *If yes,*
      1. Which further treatment(s) have you referred patients to for their depression outside of your office? (Select all that apply)
         1. Repetitive Transcranial Magnetic Stimulation (rTMS)
         2. Psychotherapy
         3. Pharmacotherapy
         4. Electroconvulsive Therapy (ECT)
         5. Other (please specify)
      2. *If select rTMS in question 2.i*
         1. How simple was the rTMS referral procedure (finding a clinic, submitting the paperwork)? *(Will answer on a scale of 1-10 with 1 being Extremely Difficult and 10 being Extremely Simple)*
         2. How accepting were your patients of the prospect of receiving rTMS treatment? *(Will answer on a scale of 1-10 with 1 being Extremely Accepting and 10 being Very Unaccepting)*
         3. Would you refer someone to receive rTMS treatment again?
            1. *If no,*

What prevents you from referring someone to rTMS again? (Select all that apply)

It's effectiveness

Safety concerns

Long waitlists

Tedious referral process

Patient acceptance

Access to treatment

Time-consuming treatment

Cost

Other (please specify)

- - - - 1. *If yes,*

What encourages you to refer someone to rTMS again? (Select all that apply)

It’s effectiveness

Limited side effects

Simple referral process

Patient acceptance

Easily accessible

Time efficient treatment

Cost

Other (please specify)

- - - 1. Would you refer someone with peripartum depression to receive rTMS treatment?
         1. *If no,*

What prevents you from referring a person with peripartum depression to receive rTMS? (Select all that apply)

It's effectiveness

Safety concerns

Long waitlists

Tedious referral process

Patient acceptance

Access to treatment

Time-consuming treatment

Cost

Other (please specify)

- - - - 1. If yes,

What encourages you to refer a person with peripartum depression to receive rTMS? (Select all that apply)

It’s effectiveness

Limited side effects

Simple referral process

Patient acceptance

Easily accessible

Time efficient treatment

Cost

Other (please specify)

1. *If they do not select rTMS in question 1 or 2*
   1. Are you aware of the existence of repetitive transcranial magnetic stimulation (rTMS) as a treatment option for depression? *(answer options: Yes or No)*
      1. *If yes,*
         1. Is there an rTMS program in the province you work in? *(answer options: Yes, No, Unsure)*
            1. If yes,

Is there an rTMS program in the city you work in? *(answer options: Yes, No, Unsure)*

*If yes,*

At your place of work, is there an onsite clinical rTMS program? *(answer options: Yes, No, Unsure)*

- - - 1. Have you received information about rTMS treatment? *(answer options: Yes or No)*
         1. *If no,*

Are you aware that rTMS is approved by Health Canada as a treatment for depression *(answer options: Yes or No)*

What information do you think is important for mental health professionals to know about rTMS as a treatment for peripartum depression? (select all that apply)

What it is

What it’s used for

How it is delivered

Who can and can't be treated with it (contraindications)

It’s effectiveness

Side effects

Required time commitment

Its cost

Is it covered by health insurance

How to access it

Other (please specify)

Would you attend an information session on rTMS applications if offered? *(answer options: Yes or No)*

1. *If answered rTMS in question 1 or 2, or answered yes to question 3.2*
   1. Are you aware that rTMS is approved by Health Canada as a treatment for depression? *(answer options: Yes or No)*
   2. What type of information have you received regarding rTMS? (Select all that apply)
      1. Theory
      2. Parameter settings
      3. Indications
      4. Contraindications
      5. Side effects
      6. Other (please specify)
   3. Are you aware of how to access rTMS treatment? *(answer options: Yes or No)*

1. Indicate your level of agreement to the following statements: (answers: 1= strongly disagree, 2= disagree, 3= neutral/unsure, 4= agree, 5= strongly agree)
   1. Knowing that rTMS is an available treatment option is important.
   2. Safety is a main concern when prescribing/providing treatment for patients with peripartum depression.
   3. rTMS is a safe and effective treatment for peripartum depression.

*Recommendations*

1. What recommendations do you have regarding rTMS treatment for peripartum depression? (Select all that apply)
   1. rTMS should be covered by healthcare
   2. Increase the amount of rTMS research
   3. Provide more formal training in rTMS theory and application to mental health professionals
   4. Increase public awareness on rTMS
   5. Increase the availability of rTMS treatment devices

1. Do you have any additional recommendations regarding rTMS treatment for peripartum depression not mentioned in the list above? Please Specify

_ _ _ _ _ _ _ _ _ _ _ _ _ _ _ _ _ _ _ _ _ _ _ _ _ _ _ _ _ _ _ _ _ _ _ _ _ _ _ _ _ _ _ _ _ _ _ _ _ _

1. How did you hear about this survey?

a. Poster at a clinic

b. Poster at a University

c. Online information

d. Email

e. Social Media

f. Other (please specify)

NOTE: Following any option selection in question 1, participants will be asked to specify the organization name or clinic that informed them about the survey. (They do not have to answer this follow-up question if they don’t want to)

As part of this research project, the research team is also conducting focus groups and interviews to ask individuals about their experiences, knowledge, and attitudes towards rTMS as a treatment for peripartum depression.

Would you like us to contact you in the future about possibly participating in a focus group or interview? Answering "yes" does not create any obligation on your part. This simply gives us permission to contact you.

1. Yes
2. No

*If yes, provide a link to the contact form.*

To ensure the anonymous nature of your survey is maintained please provide us with your contact information through the below link.

__________________________________________________________________________

**Survey 2: Patients with peripartum depression or their family**

*Demographic*

1. What gender do you currently identify as?
   1. Woman
   2. Man
   3. Non-binary
   4. Transgender
   5. Prefer not to say
   6. Other (please specify)
2. What is your age (in years)?
3. Please specify your ethnicity. (Select all that apply)
   1. Asian/ Pacific Islander
   2. Latino or Hispanic
   3. African/Caribbean/Black Canadian
   4. Caucasian
   5. First Nations/Metis/Inuit
   6. Middle Eastern
   7. Unknown
   8. Prefer not to say
   9. Other (please specify)
4. What are the first three digits of your postal code?

__________________________________________________________________________

*Experience*

1. Depression that begins during pregnancy or shortly after giving birth (within 4 weeks) is referred to as peripartum depression.
2. Have you ever felt depressed while pregnant or within a few weeks after giving birth? *(answer options: Yes or No)*
3. Have you ever been diagnosed with peripartum depression? *(answer options: Yes or No)*
   1. *If yes to questions 2 or 3,*
      1. Which of the following symptoms have you experienced while pregnant or within a few weeks after giving birth ? (select all that apply)
         1. Sad mood most of the day, almost every day
         2. Reduced interest in previously enjoyed activities
         3. Weight change (gain or loss)
         4. Sleep pattern changes (too much or too little sleep)
         5. Feeling restless
         6. Feeling slowed down or tired
         7. Feeling worthless
         8. Feeling inappropriate guilt
         9. Can’t concentrate
         10. Frequent thoughts of death
         11. Suicidal thoughts without a plan
         12. Suicidal thoughts with a plan
         13. Suicide attempt
         14. Other (please specify)
      2. When did your depressive symptoms begin?
         1. Before pregnancy
         2. During pregnancy
         3. Within 4 weeks after birth
         4. After giving birth by more than 4 weeks, but less than 1 year
         5. Other (please specify)
   2. If yes to question 3
      1. Did you receive treatment for your diagnosed peripartum depression? *(answer options: Yes or No)*
         1. *If yes,*
            1. What treatment(s) were you prescribed to treat your depression? (Select all that apply)

Repetitive Transcranial Magnetic Stimulation (rTMS)

Talk Therapy

Medication

Shock therapy (electroconvulsive therapy; ECT)

Self-treated (please specify)

Other (please specify)

*If choose rTMS,*

How helpful was rTMS in your case? *(Will answer on a scale of 1-10 with 1 being ‘Not Helpful At all’ and 10 being ‘Very Helpful’)*

How do you rate your overall experience with rTMS? *(Will answer on a scale of 1-10 with 1 being dreadful and 10 being excellent)*

Would you accept to receive rTMS treatment again? *(answer options: Yes or No)*

*If no,* “What is preventing you from receiving rTMS again? (Select all that apply)”

Did not improve symptoms

Side effects were intolerable

Risk of seizure or manic episodes

Treatment center is too far away

Long waitlist

Can’t access childcare

Time Consuming

Cost

Other (please specify)

*If yes,* “What is encouraging you to receive rTMS again? (Select all that apply)”

Improved symptoms

Side effects were tolerable

Easily accessible

Cost-efficient

Other (please specify)

- 1. If yes to question 2
     1. Did you seek any help for your depression? (answer options: yes or no)
        1. If no,
           1. What prevented you from getting help for your depression? (Select all that apply)

Couldn’t access a doctor

Couldn’t receive a diagnosis

Couldn’t afford treatment

Symptoms improved on their own

Self-treated (please specify)

Other (please specify)

- - - 1. If yes,
         1. Who did you seek help from? (Select all that apply)

Doctor

Psychiatrist

Family

Friend

Religious leader

Other (please specify)

- - - - 1. Did you receive any treatment for your depression? (Answer options: yes or no)

If no,

What prevented you from receiving treatment for your depression?

Couldn’t receive a diagnosis

Couldn’t afford treatment

Couldn’t access treatment

Symptoms improved on their own

Self-treated (please specify)

Other (please specify)

If yes,

What treatment(s) were you prescribed to treat your depression? (Select all that apply)

Repetitive Transcranial Magnetic Stimulation (rTMS)

Talk Therapy

Medication

Shock therapy (electroconvulsive therapy; ECT)

Self-treated (please specify)

Other (please specify)

*If choose rTMS,*

How helpful was rTMS in your case? *(Will answer on a scale of 1-10 with 1 being ‘Not Helpful At all’ and 10 being ‘Very Helpful’)*

How do you rate your overall experience with rTMS? *(Will answer on a scale of 1-10 with 1 being dreadful and 10 being excellent)*

Would you accept to receive rTMS treatment again? *(answer options: Yes or No)*

*If no,* “What is preventing you from receiving rTMS again? (Select all that apply)”

Did not improve symptoms

Side effects were intolerable

Risk of seizure or manic episodes

Treatment center is too far away

Long waitlist

Can’t access childcare

Time Consuming

Cost

Other (please specify)

*If yes,* “What is encouraging you to receive rTMS again? (Select all that apply)”

Improved symptoms

Side effects were tolerable

Easily accessible

Cost-efficient

Other (please specify)

1. Among your family, has anyone ever been diagnosed with peripartum depression? *(answer options: Yes, No Unsure)*
   1. *If yes,*
      1. When did their depressive symptoms begin?
         1. Before pregnancy
         2. During pregnancy
         3. Within 4 weeks after birth
         4. After giving birth by more than 4 weeks, but less than 1 year
         5. Unsure
         6. Other (please specify)
      2. Among your family, has anyone ever received treatment for their diagnosed peripartum depression? *(answer options: Yes, No Unsure)*
         1. *If yes,*
            1. What treatment(s) did they receive to treat their depression? (Select all that apply)

Repetitive Transcranial Magnetic Stimulation (rTMS)

Talk Therapy

Medication

Shock therapy (electroconvulsive therapy; ECT)

Unsure

Other (please specify)

*If choose rTMS,*

How helpful was rTMS in their case? *(Will answer on a scale of 1-10 with 1 being ‘Not Helpful At all’ and 10 being ‘Very Helpful’)*

1. *If they did not choose rTMS in either question 2 or 3*
   1. Are you aware of the existence of repetitive transcranial magnetic stimulation (rTMS) as a treatment for depression? *(answer options: Yes or No)*
      1. If suggested by a doctor, would you consider rTMS as an appropriate treatment for women with peripartum depression? *(answer options: Yes, No, or I don’t have enough information to answer this question)*
         1. *If no*
            1. Why do you consider rTMS an inappropriate treatment for women with peripartum depression? (Select all that apply)

Treatment center is too far away

Long waitlist

Can’t access childcare

Time consuming

Cost

Potential side effects

Unsure

Other (please specify)

- - - 1. *If yes*
         1. Why do you consider rTMS an appropriate treatment for women with peripartum depression? (Select all that apply)

Effective treatment

Tolerable side effects

Easily accessible

Cost-efficient

Recommended by doctor

Unsure

Other (please specify)

- 1. Are you aware that rTMS is approved by Health Canada as a treatment for depression? *(answer options: Yes or No)*

1. Repetitive transcranial magnetic stimulation (rTMS) is a safe and effective treatment for depression that does not require you to take any medication. Most often, it has been used to successfully treat cases of depression that have not responded to two or more other treatments (i.e., medication) in the past. RTMS treatment involves a strong magnet being put on your head for a short period of time, 5 days a week for 4-6 weeks. This treatment is referral-based and takes place in a clinic.
2. If you need to access rTMS, who would you prefer to go to for a referral? (Select all that apply)
   1. A family doctor
   2. A therapist
   3. A pharmacist
   4. A psychiatrist
   5. A neurologist
   6. A psychologist
   7. Obstetrician
   8. A relative, a friend or a colleague
   9. A religious leader
   10. Unsure
   11. Other (please specify)
3. Do you think women with peripartum depression require professional treatment for their depression? *(answer options: Yes, No, Unsure)*
4. If you or someone you know needs mental health treatment for peripartum depression, which treatment option would you prefer? (Select all that apply)
   1. Talk therapy
   2. Medication
   3. Repetitive transcranial Magnetic Stimulation (rTMS)
   4. Shock therapy (electroconvulsive therapy; ECT)
   5. None
   6. I don't know
   7. Other (please specify)
5. If you have depression, would you be willing to receive rTMS treatment in a clinic for 20 minutes, 5 days a week for 4-6 weeks? *(answer options: Yes, No, Unsure)*
   1. Please explain your answer (**Completion of this question is optional**)

Recommendations

1. *If choose rTMS in question 2 or 3 (under experience heading)*
   1. In your opinion, what barriers may prevent someone from accessing rTMS treatment? (Select all that apply)
      1. Do not know where or how to access it
      2. No transportation
      3. No childcare
      4. No family/partner support
      5. Work schedule
      6. Treatment cost
      7. Doesn't trust the mental health system
      8. Do not know if the treatment is effective
      9. Hope that they will feel better on their own
      10. Other (please specify)
   2. What recommendations do you have regarding rTMS treatment for peripartum depression? (Select all that apply)
      1. rTMS should be covered by healthcare
      2. Increase amount of rTMS research
      3. Provide more formal training in rTMS theory and application to mental health professionals
      4. Increase public awareness on rTMS
      5. Increase the availability of rTMS treatment devices
      6. Other (please specify)
2. *If did not select rtms in questions 2 or 3 (under experience heading)*
   1. Regarding rTMS treatment, what information would you like to receive? (Select all that apply)
      1. What it is and how its works
      2. What it’s used for
      3. How it’s delivered
      4. Who can and can't be treated with it
      5. How effective is it
      6. What are the side effects
      7. Required time commitment
      8. Its cost
      9. Is it covered by health insurance
      10. How to access it
      11. Other (please specify)

_ _ _ _ _ _ _ _ _ _ _ _ _ _ _ _ _ _ _ _ _ _ _ _ _ _ _ _ _ _ _ _ _ _ _ _ _ _ _ _ _ _ _ _ _ _ _ _ _ _

2. How did you hear about this survey?

a. Poster at a clinic

b. Poster at a University

c. Online information

d. Email

e. Social Media

f. Other (please specify)

NOTE: Following any option selection in question 1, participants will be asked to specify the organization name or clinic that informed them about the survey. (They do not have to answer this follow-up question if they don’t want to)

As part of this research project, the research team is also conducting focus groups and interviews to ask individuals about their experiences, knowledge, and attitudes towards rTMS as a treatment for peripartum depression.

Would you like us to contact you in the future about possibly participating in a focus group or interview? Answering "yes" does not create any obligation on your part. This simply gives us permission to contact you.

1. Yes
2. No

*If yes, provide a link to the contact form.*

To ensure the anonymous nature of your survey is maintained please provide us with your contact information through the below link.
