# Supplementary material for: Treating maternal depression: understanding barriers and facilitators to repetitive transcranial magnetic stimulation treatment in Canada-a protocol
Source: Front Psychiatry. 2023 Jul 27;14:1143403. doi: 10.3389/fpsyt.2023.1143403 (PMC10412871; doi:10.3389/fpsyt.2023.1143403)
Supplement: Supplementary file 2 [file Data_Sheet_2.docx]

**Semi-Structured Interview Guiding Questions**

**1. Health Professionals**

1. Have you ever provided professional services or treatments to people suffering from peripartum depression? If yes, may you please describe your experience providing services to this population.
2. Are there any special considerations you have when treating patients with peripartum depression.
3. Are there any barriers you can identify that may prevent someone with peripartum depression from seeking care?
4. Are there any barriers you can identify that may prevent someone with peripartum depression from receiving care?
5. Are you aware of a treatment option called repetitive transcranial magnetic stimulation (rTMS).
   1. If yes,
6. What do you know about rTMS
7. How do you feel about rTMS
8. Have you ever referred someone to receive rTMS treatment
9. Would you refer/recommend rTMS treatment to someone with peripartum depression
10. What barriers may prevent someone from receiving rTMS treatment
    1. If no,
       1. Repetitive transcranial magnetic stimulation (rTMS) is a safe and effective treatment for depression that does not require you to take any medication. Most often, it has been used to successfully treat cases of depression that have not responded to two or more other treatments (i.e., medication) in the past. RTMS treatment involves a strong magnet being put on your head for 20 minutes, 5 days a week for 4-6 weeks. This treatment is referral-based and takes place in a clinic.
       2. Would you refer/recommend rTMS treatment to someone with peripartum depression?
       3. What barriers may prevent someone from receiving rTMS treatment
11. What steps are needed to improve access to mental health treatment in patients with peripartum depression?
12. The main purpose of this research is to identify barriers that may be preventing someone with peripartum depression from accessing treatment in general and rTMS treatment specifically. We hope to use this information to then design a future study aimed at improving the identified barriers. Would you be interested in participating in the design of this future study sometime during the summer? Your participation can be in the form of focus group participation, or written feedback over email.

**2. Patients with Current/Previous Peripartum Depression**

1. Have you ever experienced depressive symptoms while pregnant or shortly after giving birth? If yes, may you please describe your experience.
2. Did you receive treatment?
   1. Yes= what treatments did you receive, how was your experience
   2. No= did you seek treatment, what barriers prevented you from receiving treatment.
3. What considerations do you have before accessing treatment while pregnant or breastfeeding?
4. What factors would make the treatment access process easier.
5. Are you aware of a treatment option called repetitive transcranial magnetic stimulation (rTMS).
   1. If yes,
      1. What do you know about rTMS
      2. How do you feel about rTMS
      3. Have you, or someone you know ever been treated with rTMS
      4. What barriers may prevent someone from receiving rTMS treatment
   2. If no,
      1. Repetitive transcranial magnetic stimulation (rTMS) is a safe and effective treatment for depression that does not require you to take any medication. Most often, it has been used to successfully treat cases of depression that have not responded to two or more other treatments (i.e., medication) in the past. RTMS treatment involves a strong magnet being put on your head for 20 minutes, 5 days a week for 4-6 weeks. This treatment is referral-based and takes place in a clinic.
      2. Would you accept to receive rTMS treatment for depressive symptoms while pregnant or breastfeeding?
      3. What barriers may prevent someone from receiving rTMS treatment
6. What steps are needed to improve access to mental health treatment in patients with depression while pregnant or breastfeeding in your community?
7. The main purpose of this research is to identify barriers that may be preventing someone with depression while pregnant or breastfeeding from accessing treatment in general and rTMS treatment specifically. We hope to use this information to then design a future study aimed at improving the identified barriers. Would you be interested in participating in the design of this future study sometime during the summer? Your participation can be in the form of focus group participation, or written feedback over email.
